# Gut Microbiome Alterations in Alzheimer’s Disease: A Shotgun Metagenomic Case–Control Study

**DOI:** 10.3390/ijms27219543

**Published:** 2026-10-25

**Authors:** Argul Issilbayeva, Samat Kozhakhmetov, Almagul Kushugulova, Sholpan Askarova, Aiym Kaiyrlykyzy, Dilyar Tuigunov, Oksana Dolmatova, Zhibek Zholdassova, Yerzhan Omarov, Talgat Nurgozhin

**Affiliations:** 1 Innovative Center ArtScience, Astana Z11F5A9, Kazakhstan; 2 Laboratory of Microbiome, Center for Life Sciences, National Laboratory Astana, Nazarbayev University, Astana Z05H0P9, Kazakhstan; skozhakhmetov@nu.edu.kz (S.K.); akushugulova@nu.edu.kz (A.K.); 3 Laboratory of Bioengineering and Regenerative Medicine, Center for Life Sciences, National Laboratory Astana, Nazarbayev University, Astana Z05H0P9, Kazakhstan; shaskarova@nu.edu.kz (S.A.); aiym.kaiyrlykyzy@nu.edu.kz (A.K.); 4 Laboratory of Food Biotechnology and Specialty Food Products, Kazakh Academy of Nutrition, Almaty A05D9M8, Kazakhstan; dilyar117@gmail.com (D.T.); oksana.dolmatova@yahoo.com (O.D.); yerzhan_omarov@mail.ru (Y.O.); tnurgozhin@kaznmu.kz (T.N.); 5 Neurosis and Alzheimer’s Disease Treatment Center, Almaty A05E1P7, Kazakhstan; zhibek_zholdas@mail.ru; 6 School of General Medicine, Asfendiyarov Kazakh National Medical University, Almaty A05H2A6, Kazakhstan

**Keywords:** gut microbiome, Alzheimer’s disease, shotgun metagenomics, case–control study, dysbiosis, adiponectin, Central Asia, Kazakhstan

## Abstract

The gut microbiome has been proposed as a contributor to Alzheimer’s disease (AD) pathogenesis via the microbiota–gut–brain axis, but findings across cohorts are inconsistent and scarce data exist from Central Asian populations. We used shotgun metagenomic sequencing to compare the gut microbiome of AD patients and cognitively healthy controls from Kazakhstan. In a cross-sectional case–control study (35 AD, 39 Control), we profiled taxonomic and functional (HUMAnN pathway) composition, and compared alpha/beta diversity, individual taxa and pathways, and their association with clinical and demographic variables (Mann–Whitney U test, PERMANOVA/PERMDISP, OLS regression, all BH-FDR corrected, sensitivity analysis using ANCOM-BC2), with sensitivity analyses adjusting for age and BMI and applying Bonferroni correction. Within the AD group, circulating adiponectin—measured only in this group—was examined for associations with microbiome features in an exploratory analysis. Alpha diversity was lower in AD (*q* < 0.05). Community composition also separated by group on PERMANOVA (R^2^ = 8.6%, *p* = 0.001), and this separation held after adjusting for age and BMI. A total of 178 taxa differed significantly between groups (BH-FDR; 37 were also Bonferroni-significant), most prominently depletion of *Faecalibacterium* and enrichment of *Eisenbergiella* and *Desulfovibrio* in AD; 100 pathways differed significantly (20 Bonferroni-significant). Three clinical/demographic variables (total bilirubin, atherogenic index, BMI) were associated with specific taxa or pathways. Within the AD group, adiponectin correlated negatively with the pathway assimilatory sulfate reduction IV (rho = −0.60, *q* = 0.015). AD in this Central Asian cohort was associated with a less diverse, compositionally distinct gut microbiome, with depletion of butyrate-producing Firmicutes and enrichment of sulfate-reducing taxa, alongside other taxa whose direction of association differed from prior reports.

## 1. Introduction

The gut microbiome is a plausible contributor to Alzheimer’s disease (AD) pathogenesis, but it is not yet well established. Human studies generally report altered community structure and lower species richness in AD [1]. The leading mechanistic model is a microbiota–gut–brain axis, alteration of which may affect intestinal permeability, systemic inflammation, microglial activation, and amyloid/tau-related pathology. This mechanism is supported by animal and in vitro evidence; it has not been established in human clinical studies, and the human associations reported to date between microbial composition, inflammation, and AD pathology do not themselves establish causality.

The existing taxonomic signal reported for AD is inconsistent: a recent systematic review and meta-analysis found the same genera reported as increased in some cohorts and decreased in others, depending on the population studied [2]. A recent shotgun metagenomic study spanning AD pathological stages found both taxonomic and gene-family-level shifts that tracked disease severity [3], and functional gut microbiome features have separately been linked to markers of AD pathology [4]. One of the limitations of the field is geographic: gut microbiome cohorts are mostly drawn from North America, Europe, and East Asia, while Central and Southern Asia contribute under 2% of public microbiome samples [5]. Within Kazakhstan specifically, two prior case–control studies from Astana have reported gut dysbiosis in AD, both using 16S rRNA amplicon sequencing [6,7]. Both prior Kazakhstani studies relied on 16S rRNA amplicon sequencing, which resolves taxa only to the genus level and cannot characterize functional pathways; shotgun metagenomics provides species-level resolution and direct functional profiling, neither of which are available from the existing AD microbiome literature in this country. We have previously reported oral microbiome shifts associated with AD [8]. The present study extends this work to a distinct cohort recruited in Almaty, geographically separate from the prior Astana-based studies, and, to our knowledge, is among the scarce shotgun metagenomic case–control studies on AD reported from a Kazakhstani or broader Central Asian cohort, despite recent evidence that gut microbial composition in this population differs from previously studied groups [9].

In this study, we aimed to characterize the gut microbiome of AD patients relative to cognitively healthy controls in a Kazakhstani cohort using shotgun metagenomic sequencing. This provides a shotgun metagenomic data point from an underrepresented population; distinguishing which AD-associated microbial signals generalize across populations from those that are cohort- or region-specific will require direct cross-cohort comparisons, which are beyond the scope of the present study.

## 2. Results

The two groups were balanced by sex (25.7% vs. 23.1% male, *p* = 1.0000) and did not differ significantly by ethnicity (*p* = 0.088), though AD patients were considerably older (82.0 [IQR 74.0–87.5] vs. 67.0 [61.0–71.5] years, *p* < 0.0001) and had a lower BMI (23.1 [21.7–25.2] vs. 26.5 [23.6–29.0] kg/m^2^, *p* = 0.0014). Glucose and insulin levels were significantly lower in the AD group, while remaining laboratory parameters (ALT, AST, total bilirubin, CRP, total cholesterol, HDL, LDL, triglycerides, atherogenic index) did not differ significantly between groups (Table 1). Individual age distributions are shown in Figure S1A; the groups’ interquartile ranges did not overlap, though ages converged between 70 and 80 years (13 AD, 13 Control). 

**Figure 1:**
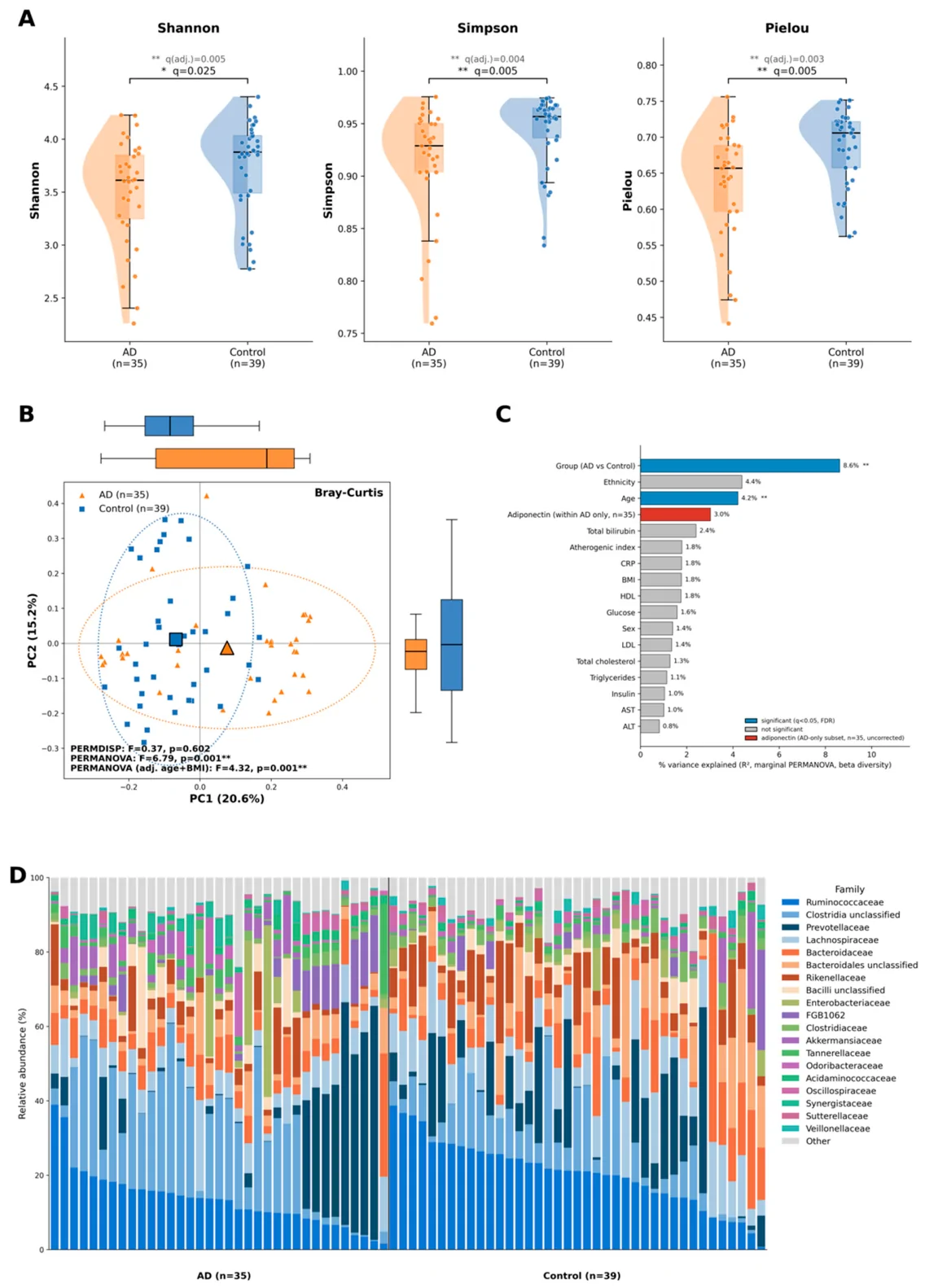
Gut microbiome diversity, composition, and association with clinical and demographic variables in Alzheimer’s disease. (**A**) Alpha diversity metrics—Shannon, Simpson, and Pielou’s evenness—in AD and Control subjects; * q < 0.05, ** q < 0.01 (BH-FDR; unadjusted and age/BMI-adjusted [q(adj.)]). (**B**) PCoA ordination based on Bray–Curtis dissimilarity, genus level, Hellinger-transformed; ** denotes PERMANOVA *p* < 0.01. (**C**) Marginal PERMANOVA screening of clinical and demographic variables for association with microbial community composition; ** denotes FDR-significant associations (q < 0.05) (**D**) Relative abundance at the family level across study groups.

Alpha diversity was consistently lower in AD relative to Control patients. Shannon (3.61 [IQR 3.25–3.85] vs. 3.88 [IQR 3.49–4.03]; *q* = 0.025), Simpson (0.93 [0.90–0.95] vs. 0.96 [0.94–0.96]; *q* = 0.005) and Pielou’s evenness (0.66 [0.60–0.69] vs. 0.71 [0.66–0.72]; *q* = 0.005) all differed between groups, and the differences held after adjusting for age and BMI (*q* = 0.005, 0.004 and 0.003, respectively; Figure 1A, Supplementary Table S1).

Community composition also separated by group. PERMANOVA on Bray–Curtis dissimilarities showed a clear group effect (F = 6.79, *p* = 0.001, R^2^ = 8.6%) that remained after adjusting for age and BMI (F = 4.32, *p* = 0.001, partial R^2^ = 5.8%; PERMDISP F = 0.37, *p* = 0.602) (Figure 1B). 

A sensitivity analysis restricted to this age-overlapping subset (*n* = 13 per group) gave a similar effect size (R^2^ = 8.5%) despite reduced power (F = 2.23, *p* = 0.013), and 98% of the 178 differentially abundant taxa and 99% of the 100 pathways kept the same direction of effect (Tables S1–S3).

Screening the available clinical and demographic variables against beta diversity, only diagnosis and age remained significant (R^2^ = 8.6%, *q* = 0.008 and R^2^ = 4.2%, *q* = 0.008); none of the remaining metabolic, lipid or inflammatory markers showed any relationship. Adiponectin, available only in the AD group, was not associated with its microbiome composition either (R^2^ = 3.0%, *p* = 0.350) (Figure 1C, Supplementary Table S1).

Family-level profiles differed visibly between the two groups (Figure 1D); the dominant families, with their prevalence and typical abundance, are listed in Supplementary Table S1.

**Figure 2:**
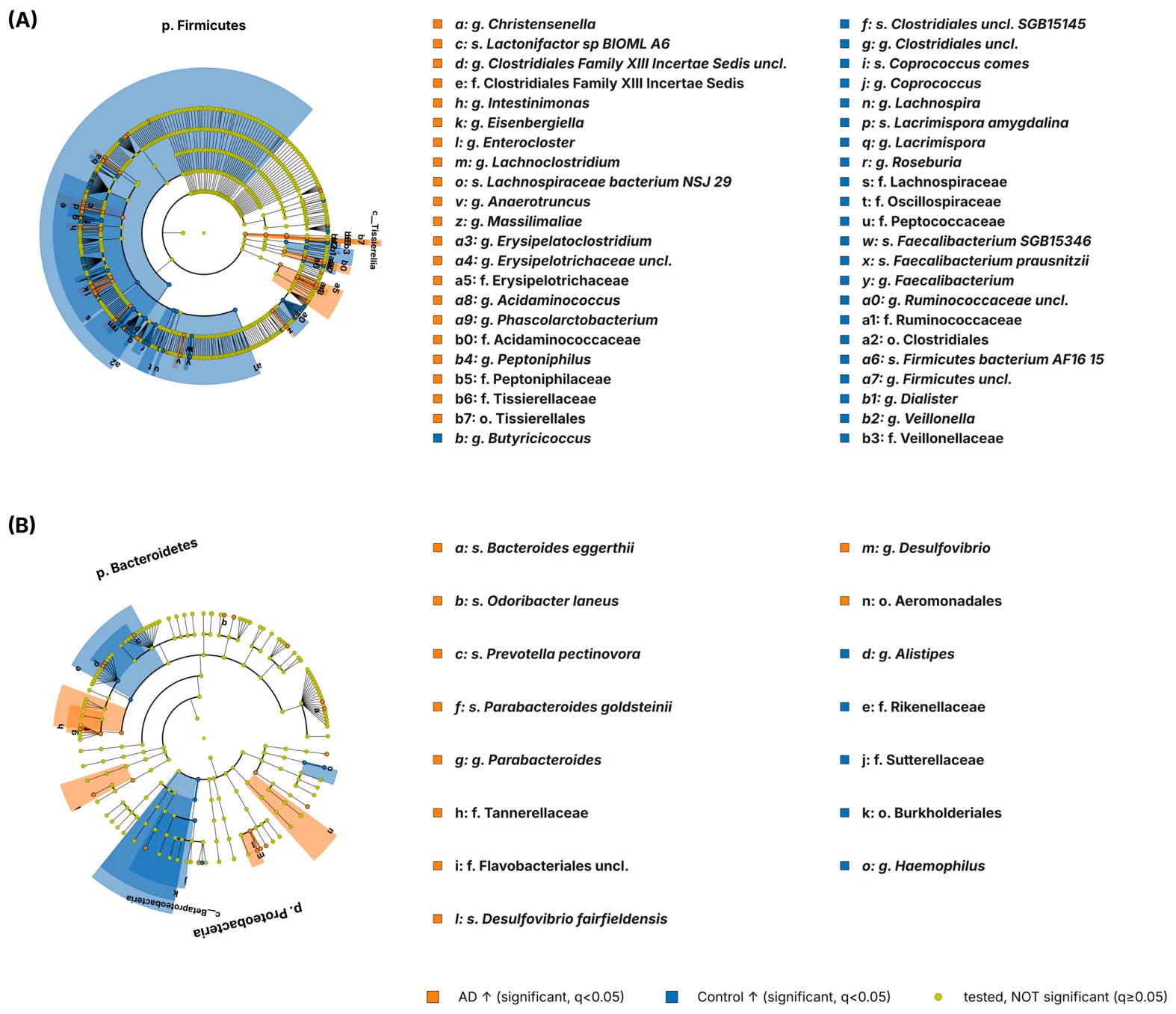
Cladogram of differentially abundant gut microbial taxa (phylum to species level) between AD patients and cognitively healthy controls, rendered using the LEfSe plotting utility (repurposed for tree visualization only, as described in Section 4); taxa were identified by two-sided Mann–Whitney U test with BH-FDR correction (*q* < 0.05). (**A**) Significant taxa within phylum Firmicutes. (**B**) Significant taxa within phyla Bacteroidetes and Proteobacteria. Node color indicates the direction of enrichment (orange = AD, blue = Control). Lettered markers identify taxa down to genus level, plus the most significant species per panel (top 8 in (**A**), top 5 in (**B**)).

Comparison of AD and Control gut microbiomes identified 178 of 601 tested clades that differed significantly in relative abundance between AD patients and controls (Mann–Whitney U test, *q* < 0.05, BH-FDR). Of these, 120 belonged to phylum Firmicutes (Figure 2A), and 30 to phyla Bacteroidetes and Proteobacteria combined (Figure 2B); the remaining 28 significant taxa, spanning seven additional phyla (*Actinobacteria*, *Candidatus Saccharibacteria*, *Candidatus Thermoplasmatota*, *Euryarchaeota*, *Fusobacteria*, *Lentisphaerae*, and *Synergistetes*), are reported in Supplementary Table S2. After adjustment for age and BMI (OLS on arcsine-square-root-transformed relative abundances), 81 clades were significant (*q*_adj < 0.05), including 73 overlapping with the unadjusted (primary) analysis and eight significant only after adjustment. Supplementary Table S2 flags whether each of the 178 taxa retained significance after adjustment (*q*_adj < 0.05). *Faecalibacterium*, *Eisenbergiella*, and *Desulfovibrio* remained significant with stable effect size in all three nested models (Supplementary Table S1).

Within Firmicutes (Figure 2A), the strongest signal was a nearly four-fold depletion of *Faecalibacterium* in AD relative to controls (1.50% vs. 5.72%; *q* = 1.1 × 10^−5^), consistent with reduced butyrate-producing capacity. Order *Clostridiales* was also depleted overall in AD (24.47% vs. 36.06%; *q* = 4.9 × 10^−5^), together with genera *Coprococcus* (*q* = 1.0 × 10^−3^) and *Lachnospira* (*q* = 2.3 × 10^−3^), both butyrate producers within family *Lachnospiraceae*. Conversely, genus *Eisenbergiella* was strongly enriched in AD (0.42% vs. 0.01%; log2FC = 5.14; *q* = 4.9 × 10^−5^).

Within *Bacteroidetes* and *Proteobacteria* (Figure 2B), genus *Alistipes* was depleted in AD (2.52% vs. 6.47%; *q* = 0.012), as was family *Rikenellaceae* (*q* = 5.9 × 10^−3^). In contrast, order *Aeromonadales* (*q* = 2.3 × 10^−3^) and genus *Desulfovibrio* (0.33% vs. 0.18%; *q* = 3.1 × 10^−3^) were both enriched in AD.

Because the 601 tested clades span six nested taxonomic ranks and are therefore not all statistically independent, we additionally checked how many findings survive the substantially more conservative Bonferroni correction. Of the 178 BH-FDR-significant taxa, 37 (21%) also survived Bonferroni correction (α = 0.05/601 = 8.3 × 10^−5^), including both key genus-level findings highlighted above (*Faecalibacterium*, *p* = 8.9 × 10^−8^; *Eisenbergiella*, *p* = 1.2 × 10^−6^) and the species *Desulfovibrio fairfieldensis* (*p* = 3.6 × 10^−5^); the genus *Desulfovibrio* itself narrowly missed this stricter threshold (*p* = 3.1 × 10^−4^) while remaining significant by FDR (*q* = 0.003). Of the 100 BH-FDR-significant pathways, 20 (20%) survived Bonferroni correction (α = 0.05/460 = 1.1 × 10^−4^). Several secondary taxa discussed below—including *Alistipes*, *Rikenellaceae*, *Lachnospira*, and *Aeromonadales*—are significant only by the primary FDR criterion and did not survive Bonferroni. We report BH-FDR as the primary criterion, consistent with standard practice for large-scale, correlated microbiome data [10], and present the Bonferroni counts here as a transparency check rather than a revised significance threshold. We treat genus as the primary rank, matching the taxa discussed in detail here (*Faecalibacterium*, *Eisenbergiella*, *Desulfovibrio*); results at other ranks reflect the same underlying signal at different resolution. ANCOM-BC2 confirmed 72 of the 178 primary-significant taxa (40%) unadjusted and 52 (29%) after age + BMI adjustment. Among the taxa discussed above, *Eisenbergiella* (*q* = 0.031) and *Desulfovibrio* (*q* = 0.008) were confirmed; *Faecalibacterium* (*q* = 0.234) and *Clostridiales unclassified SGB15145* (*q* = 0.279) were not, though both kept the same direction as the primary analysis. Direction agreed for 175/178 taxa overall (98%) and for all 52 ANCOM-BC2-significant taxa (100%) (Supplementary Figure S2, Tables S1 and S2).

**Figure 3:**
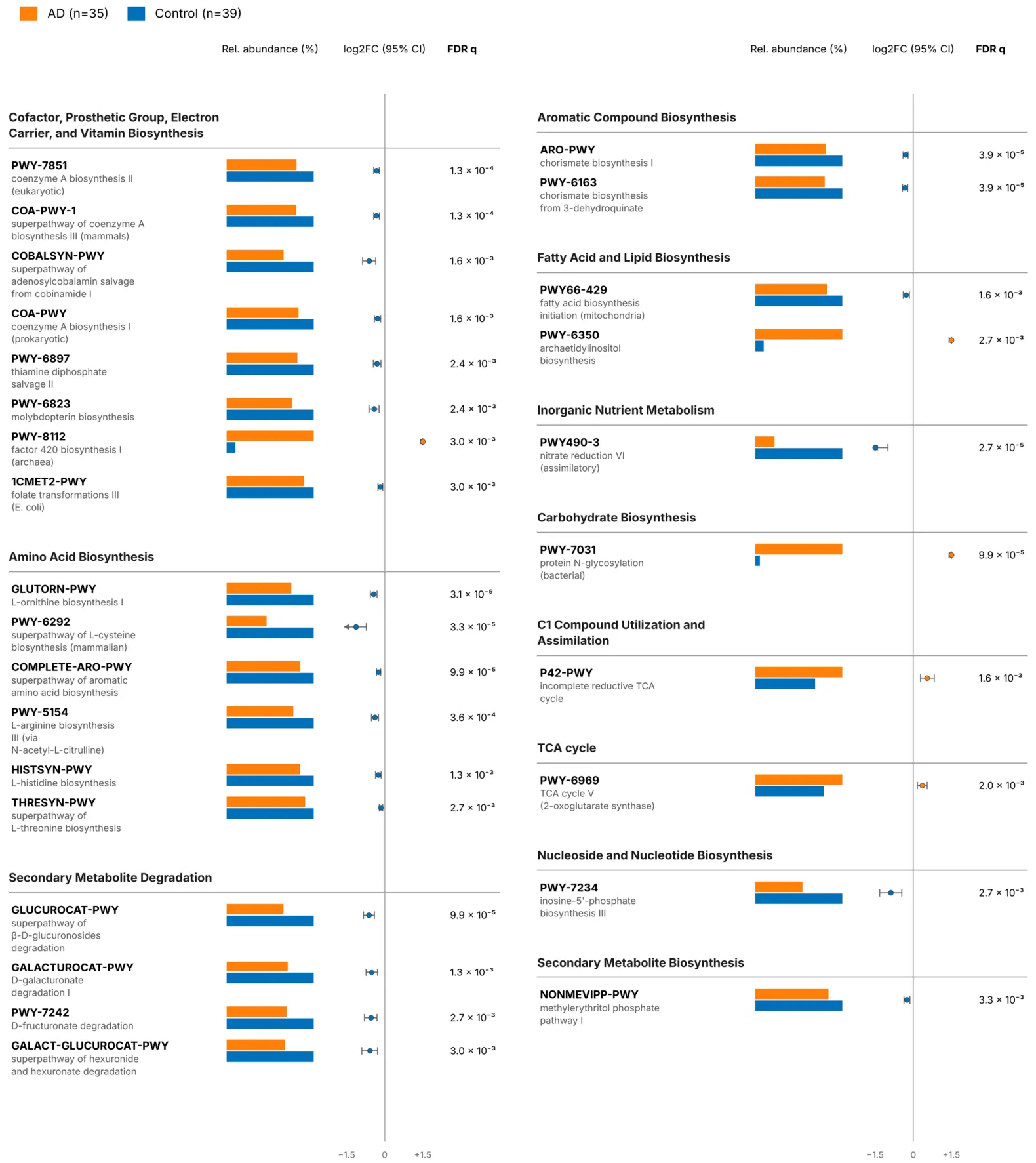
Differentially abundant metabolic pathways between AD and Control gut microbiomes. Mean relative abundance (bar panel) and log2 fold-change with 95% bootstrap confidence interval (dot-and-whisker panel) for the top 28 of 100 significant microbial metabolic pathways (Mann–Whitney U test, two-sided, BH-FDR *q* < 0.05; *n* = 460 pathways tested, ≥10% prevalence). Pathways are grouped by MetaCyc category and ranked by FDR *q* within each category; dot color indicates direction of enrichment. Confidence intervals exceeding the axis range are marked with an arrowhead (exact values in Supplementary Table S3).

At the pathway level, community-level functional profiling (HUMAnN) identified 460 metabolic pathways present in ≥10% of samples. Of these, 100 differed significantly in relative abundance between AD and Control groups (Table S3). The differentially abundant pathways spanned diverse functional categories, with cofactor/vitamin biosynthesis (eight of the top 28 pathways shown), amino acid biosynthesis (six), and secondary-metabolite degradation (four) most strongly represented among the top hits (Figure 3).

The majority of top-ranked pathways were enriched in Control samples, including aromatic amino acid/chorismate biosynthesis (ARO-PWY, PWY-6163), cysteine biosynthesis (PWY-6292, log2FC = −1.12, *q* = 3.3 × 10^−5^), and, most strongly, nitrate reduction (PWY490-3, log2FC = −2.10, *q* = 2.7 × 10^−5^). A smaller set was enriched in AD, most prominently protein *n*-glycosylation (PWY-7031, log2FC = 4.07, *q* = 9.9 × 10^−5^), archaetidylinositol biosynthesis (PWY-6350), and factor 420 biosynthesis (PWY-8112)—the latter two characterized by near-absence in Control samples and correspondingly wide confidence intervals. ARO-PWY, PWY-6163, PWY-6292, PWY490-3, and PWY-7031 also survived Bonferroni correction (Figure 3; Supplementary Table S3).

Taxon-stratified HUMAnN output confirmed that both archaea-associated pathways are driven almost entirely by *Methanobrevibacter smithii*, which accounted for a median 74% (PWY-6350) and 100% (PWY-8112) of pathway abundance in the samples where each was detected (21/35 AD samples; Supplementary Table S2). This species, together with three taxa within a second methanogen order (*Methanomassiliicoccales*), was independently identified as enriched in AD by clade-level differential abundance testing; only the *Methanomassiliicoccales* taxa remained significant after Bonferroni correction, age/BMI adjustment, and ANCOM-BC2 re-analysis (Supplementary Tables S2 and S3). Within the AD group, *Methanobrevibacter smithii*, family Methanomassiliicoccaceae, and *Methanosphaera stadtmanae* abundance each correlated negatively with the combined abundance of *Faecalibacterium*, *Coprococcus*, and *Lachnospira* (rho = −0.70, −0.68, and −0.55, respectively; *q* = 9.3 × 10^−6^, 9.3 × 10^−6^, and 5.7 × 10^−4^; BH-FDR, *n* = 35).

A total of 61 of the 100 significant pathways (61%) remained significant after adjustment for age and BMI; the other 39 did not and are more sensitive to the groups’ age/BMI difference (Table 1). Supplementary Table S3 flags this for every pathway. ARO-PWY, PWY-6163, PWY-6292, PWY490-3, PWY-6350, PWY-8112, and PWY-7031 remained significant and stable in all three nested models (Supplementary Table S1).

ANCOM-BC2 confirmed 19 of the 100 significant pathways (19%) unadjusted and two (2%) after adjustment (see Section 4)—PWY490-3 (*q* = 0.014) and PWY-7031 (*q* < 0.001). Direction agreed for 91/100 pathways overall (91%) and for both ANCOM-BC2-significant pathways (100%); the three exceptions were HISTSYN-PWY, PYRIDNUCSYN-PWY, and PWY-6385 (Supplementary Figure S2, Tables S1 and S3).

**Figure 4:**
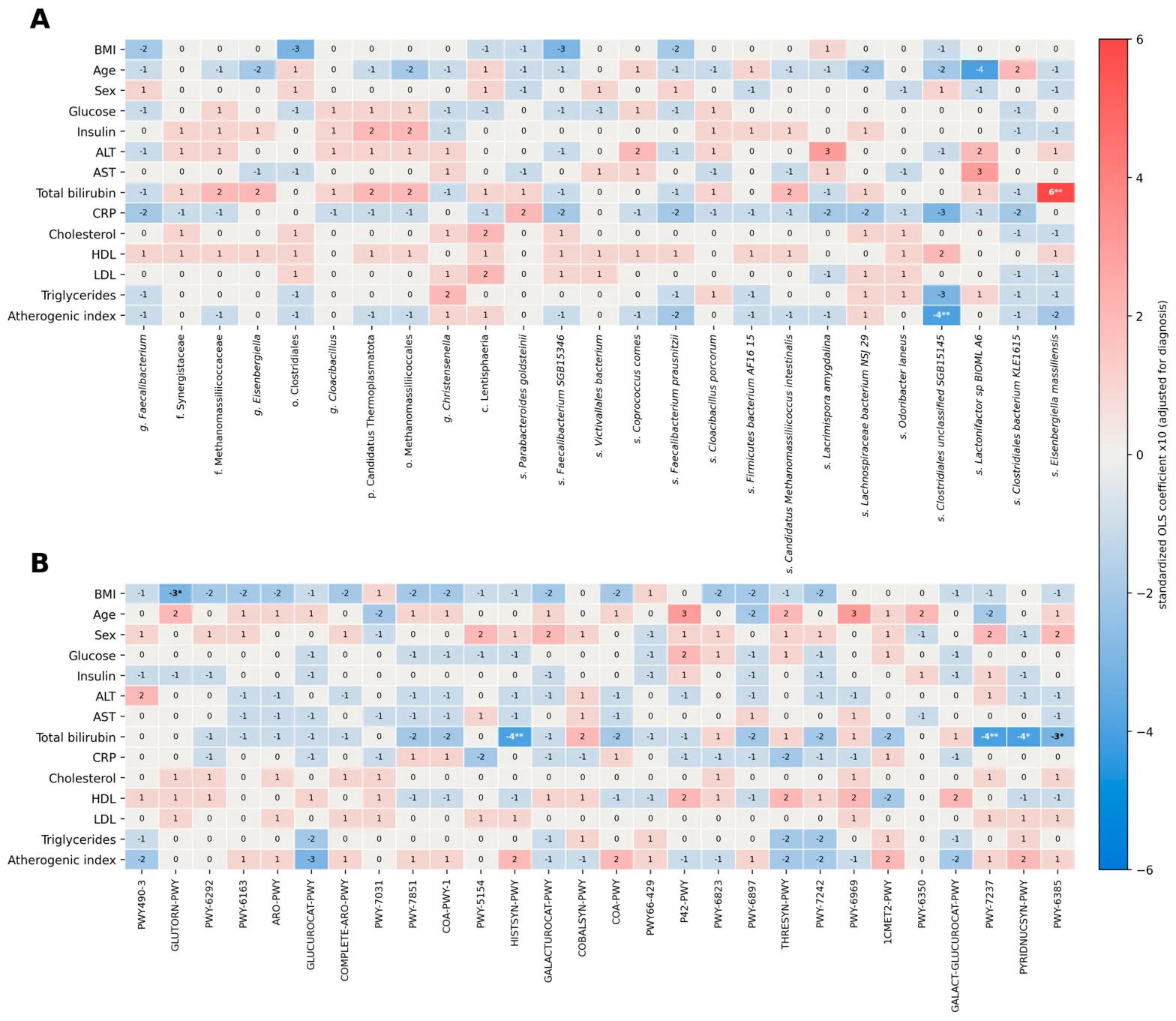
(**A**) Standardized OLS regression coefficients (×10) for 14 clinical/demographic parameters (BMI, age, sex, glucose, insulin, ALT, AST, total bilirubin, CRP, total cholesterol, HDL, LDL, triglycerides, atherogenic index) against the relative abundance of 25 taxa selected from the AD-vs-Control cladogram (Figure 2). (**B**) The same 14 parameters against 28 metabolic pathways selected from the AD-vs-Control pathway analysis (Figure 3). Full regression statistics (*n*, coefficient, *p*-value, *q*-value) for all tested associations are provided in Supplementary Table S4; * *q* < 0.05, ** *q* < 0.01 (BH-FDR).

After adjustment for AD/Control diagnosis and correction for multiple testing (BH-FDR), three clinical/demographic parameters showed significant associations with specific gut microbiome features, amounting to seven significant taxon- or pathway-level associations in total (Figure 4; Supplementary Table S4). A global BH-FDR correction across all 1019 tests (Supplementary Tables S4 and S5) retained five of these eight associations: bilirubin–*Eisenbergiella massiliensis*, atherogenic index–SGB15145, bilirubin–HISTSYN-PWY, bilirubin–PWY-7237, and adiponectin–PWY1ZNC-1. Bilirubin–PYRIDNUCSYN-PWY, bilirubin–PWY-6385, and BMI–GLUTORN-PWY did not survive and are reported as exploratory.

Total bilirubin was associated with the relative abundance of *Eisenbergiella massiliensis* (standardized coefficient = 0.6, *q* = 3 × 10^−6^) and with four metabolic pathways—L-histidine biosynthesis (HISTSYN-PWY, *q* = 0.003), myo-, chiro- and scyllo-inositol degradation (PWY-7237, *q* = 0.003), NAD de novo biosynthesis I from aspartate (PYRIDNUCSYN-PWY, *q* = 0.014), and peptidoglycan biosynthesis III (PWY-6385, *q* = 0.014), all inversely related to bilirubin levels. The atherogenic index was inversely associated with an unclassified *Clostridiales* species (SGB15145, *q* = 0.006), and BMI was inversely associated with L-ornithine biosynthesis (GLUTORN-PWY, *q* = 0.039).

Serum adiponectin was measured only in the AD group (*n* = 35; not available for Control), precluding a direct between-group comparison. Given its recognized relevance to metabolic and neurodegenerative pathophysiology, we performed an exploratory analysis of its association with the gut microbiome within the AD subgroup only. At the community level, marginal PERMANOVA showed no significant association between adiponectin and overall microbiome composition (R^2^ = 3.0%, *p* = 0.35, uncorrected; tested as a separate, single-test family since it could not be included in the main 16-variable AD + Control PERMANOVA model). At the feature level, targeted Spearman correlation analysis (283 tests: five alpha-diversity metrics, 178 taxa, 100 pathways; BH-FDR corrected within each domain) identified one significant association: adiponectin correlated negatively with the pathway PWY1ZNC-1 (assimilatory sulfate reduction IV; rho = −0.60, *q* = 0.015, *n* = 35)—a pathway independently found significantly enriched in AD versus Control patients (FDR *q* = 0.041). No associations were found with alpha diversity or any of the 178 taxa examined (Supplementary Table S5 and Figure S1).

## 3. Discussion

In this shotgun metagenomic case–control study, AD patients had a gut microbiome that was both less diverse and compositionally distinct from that of cognitively healthy controls, with taxonomic and functional shifts affecting a substantial fraction of the community. Lower alpha diversity and a clear community-level separation on Bray–Curtis dissimilarity fit the broader literature on AD and cognitive impairment, although the specific taxa driving that separation vary considerably across cohorts and geographic regions [3]. Diagnosis remained the dominant correlate of community structure even after adjusting for age and BMI (partial R^2^ = 5.8%, Section 2). Age showed a significant marginal association when screened individually among 16 clinical and demographic variables (R^2^ = 4.2%, *q* = 0.008), but this association did not persist once diagnosis group and BMI were accounted for (partial R^2^ = 1.3%, *p* = 0.484, Supplementary Table S1)—consistent with age being substantially confounded with diagnosis in this cohort, rather than reflecting an independent microbiome correlate.

At the taxon level, the most consistent theme was depletion of butyrate-producing *Firmicutes* in AD. *Faecalibacterium* was the strongest signal by raw significance (*q* = 1.1 × 10^−5^), nearly four-fold lower in AD, driven predominantly by *Faecalibacterium prausnitzii* (92–96% of genus abundance), with a smaller, independently significant contribution from *Faecalibacterium SGB15346*; under ANCOM-BC2, only SGB15346 remained significant after age/BMI adjustment (*q*_adj = 1.3 × 10^−3^), while the genus and *F. prausnitzii* did not (direction concordant throughout). Depletion was also seen in order *Clostridiales* overall and in the *Lachnospiraceae* genera *Coprococcus* and *Lachnospira*. *F. prausnitzii* is among the most abundant butyrate producers in the healthy colon and has well-documented anti-inflammatory activity [11]. Its depletion, together with reduced *Lachnospiraceae*, has previously been reported in AD-type dementia. One study went as far as identifying specific *F. prausnitzii* strains as candidates for microbiome-based intervention [12], and a dementia pilot study linked reduced butyrate producers to gut barrier dysfunction and systemic inflammation [13]. In the 3 × Tg-AD mouse model, an age-dependent decline in butyrate producers has also been shown to precede and track amyloid/tau pathology and memory deficits [14]; this temporal evidence comes from an animal model, and our cross-sectional human data cannot establish whether butyrate-producer depletion plays a causal role in AD or is instead a downstream consequence of disease-related changes in diet, gut transit, or institutional care. We did not measure fecal or serum butyrate directly; depletion of butyrate-producing taxa indicates reduced genetic potential for butyrate synthesis, not a confirmed reduction in butyrate levels.

Two of our other taxonomic findings were inconsistent with current literature. Genus *Eisenbergiella* was markedly enriched in AD here (log2FC = 5.14), yet the only prior AD-relevant report we found—a genetic-correlation analysis—identified it as the strongest genetically protective genus against AD risk, the opposite direction [15]. The genus was only described in 2014, and we located only a single prior AD-relevant report; this discrepancy may reflect genuine biological complexity as much as the limited literature; a genetic liability signal need not predict a cross-sectional abundance difference. *Alistipes* and family *Rikenellaceae* showed the reverse pattern—both depleted in AD here, but enriched in an earlier, frequently cited AD gut-microbiome study [16], and *Alistipes* associations in the genetic-correlation analysis did not replicate across cohorts [15]. *Alistipes* in particular repeatedly shows this context-dependent, sometimes opposite association across diseases and cohorts—for instance, a Kazakhstani shotgun metagenomic study of nonagenarians and centenarians found it elevated among long-lived, healthy individuals [17]. Our results are in line with the broader observation that cohort- and region-specific factors, more than any single universal AD signature, explain much of the variance in reported taxa across studies [3].

Functional profiling added a further layer beyond taxonomy: pathways for aromatic amino acid biosynthesis via chorismate (ARO-PWY, PWY-6163) were among the most robustly depleted in AD. Gut microbial tryptophan metabolism, including the balance between the kynurenine and serotonin branches, has separately drawn attention as a candidate mechanism in AD [18]. We did not measure tryptophan or its downstream metabolites, and a reduction in bacterial aromatic-amino-acid biosynthetic capacity does not by itself establish altered host tryptophan availability, kynurenine flux, or serotonin metabolism; we note this literature as background only, not as evidence of a mechanistic link. Cysteine biosynthesis (PWY-6292) and nitrate reduction (PWY490-3) were likewise depleted in AD, the latter showing the strongest Control enrichment of any pathway. This pathway (nitrate reduction VI) is an assimilatory route that fixes nitrate into microbial biomass rather than releasing nitrite for host absorption, and is mechanistically distinct from the dissimilatory nitrate-to-nitrite reduction carried out mainly by oral facultative anaerobes—the step linked to systemic nitric oxide bioavailability and vascular benefit [19]; its depletion in AD gut metagenomes does not by itself support a role for reduced vascular NO generation. The cysteine-pathway result was opposite to a recent AD-risk cohort study, which found the related cysteine-and-methionine-metabolism pathway enriched, not depleted, in participants with worse cognitive performance [20]; whether this reflects cohort stage, differing pathway definitions, or genuine population-specific biology is not resolved by our data.

Pathways enriched in AD, by contrast, were dominated by two markers of archaeal metabolism—archaetidylinositol biosynthesis (PWY-6350, an archaeal membrane-lipid pathway) and factor 420 biosynthesis (PWY-8112, the methanogenesis cofactor)—alongside bacterial protein N-glycosylation (PWY-7031). Taxon-stratified output traced both archaea-associated pathways to *Methanobrevibacter smithii*, and this species was independently more abundant in AD at the taxon level (Section 2); a second, taxonomically distinct group of methanogens, order *Methanomassiliicoccales*, was also enriched in AD and showed the most robust statistical support of any archaeal finding in this study, surviving every sensitivity check applied. *Methanobrevibacter smithii* itself, and the two archaea-associated pathways, were directionally consistent but did not survive age/BMI adjustment or ANCOM-BC2 re-analysis (Section 2), so we regard the broader pattern of archaeal enrichment, rather than any single taxon or pathway, as the more defensible finding. This runs counter to a recent report that a higher abundance of *Methanobrevibacter smithii* is associated with better, not worse, executive function and inhibitory control, mediated in part through enhanced microbial butyrate production [21]. In our data the two findings were directly coupled rather than merely co-occurring: within the AD group, both *Methanobrevibacter smithii* and family *Methanomassiliicoccaceae* correlated negatively with the combined abundance of the three butyrate-producing genera discussed above (Section 2)—the opposite relationship from that prior report. Bacterial surface-protein glycosylation, studied mainly in specific commensal and pathogenic taxa, has been shown to modulate host immune recognition—for example, altered glycosylation of surface adhesins changes dendritic-cell responses to the bacterium [22]. Our cross-sectional, pathway-level data cannot resolve the mechanism behind either finding. Both could reflect a direct biological link, a coincidental co-occurrence, or a shared underlying cause. The single feature-level correlate of adiponectin identified here is an inverse relationship with assimilatory sulfate reduction (PWY1ZNC-1), and it warrants cautious interpretation. Assimilatory sulfate reduction is a broadly distributed biosynthetic pathway, carried by many gut commensals, that reduces sulfate to sulfide for cysteine synthesis. It is mechanistically distinct from dissimilatory sulfate reduction, which specialist sulfate-reducing bacteria (e.g., *Desulfovibrio* spp.) use for anaerobic respiration—the pathway previously implicated in AD risk via excess hydrogen sulfide production [23]. However, recent work shows that assimilatory sulfate reduction can itself be a quantitatively important source of luminal H_2_S and has been linked to intestinal inflammation in other disease contexts [24], so the two pathways may be less functionally distinct than their names imply. H_2_S signaling in the CNS is concentration-dependent in AD: physiological levels are proposed to be neuroprotective (e.g., via GSK-3β sulfhydration, which limits tau hyperphosphorylation), while dysregulation may be detrimental [25]. Human observational studies of adiponectin in AD report inconsistent directions of association [26], and a proposed cysteine-to-adiponectin mechanistic link via DsbA-L [27] would actually predict the opposite direction from what we observed. Given these unresolved tensions, along with the small subgroup size (*n* = 35), the absence of a Control comparison for this marker, and the fact that this is a single association among 283 tested, we treat this finding as hypothesis-generating and in need of independent replication.

This study has several limitations. The most important one is that the AD group was recruited exclusively from a long-term care facility and the Control group from community-dwelling primary-care attendees; recruitment setting, institutional diet, and polypharmacy are therefore inseparable from diagnosis in our design. The study is also cross-sectional, so no causal direction can be inferred between any microbiome feature and AD status. The groups also differed considerably in age; an age-overlap sensitivity analysis argues against this fully explaining our findings, though residual confounding cannot be excluded in a cohort this size. BMI also differed between groups and may partly reflect AD-related appetite and nutritional changes rather than a pure confounder; adjusting for it could therefore also remove some disease-related signal. Relatedly, HUMAnN-derived pathway abundances reflect microbial genetic potential inferred from community DNA, not measured metabolite levels or gene expression; our interpretation of pathway is therefore indirect, and direct metabolomic or transcriptomic validation would be needed to confirm the proposed mechanisms. The cohort is also drawn from a single country with a modest sample size (35 AD, 39 Control), which limits statistical power for subgroup and correlation analyses and limits how far these findings generalize to other populations. The institutional diet, routine, and long-term medical treatment together with unmeasured factors such as fiber intake, bowel habit, functional status, and mobility, are confounded with AD status in this design and could not be statistically separated from disease-related effects given the sample size. Detailed medication and comorbidity data were not collected in a form suitable for quantitative analysis. Polypharmacy is likely more common among institutionalized AD patients, so it could not be adjusted for and remains a potential confounder. The microbiome differences reported here should be read as associated with the clinical state of institutionalized AD patients under these conditions, not as proof of an AD-specific effect. Likewise, AD diagnosis was clinical, without CSF or PET amyloid/tau biomarkers. The diagnostic work-up also varied with neuroimaging availability: patients without neuroimaging were classified using the Hachinski score, a less reliable proxy for distinguishing vascular from neurodegenerative contributions. The distribution of dementia severity could not be reported. Some diagnostic misclassification and heterogeneity in disease stage are therefore possible, and our findings should be interpreted as relating to clinically diagnosed AD-type dementia rather than biomarker-confirmed AD. At the same time, this is, to our knowledge, among the scarce shotgun metagenomic case–control studies of AD from a Central Asian cohort, an underrepresented population in AD gut microbiome research [6]. The clinical/demographic correlation screen and the adiponectin analysis were both exploratory, restricted to a subset of already-selected features (or, for adiponectin, to the AD group alone); we present them as hypothesis-generating rather than confirmatory. Several age/BMI-adjusted models deviate from normality or homoscedasticity assumptions (Shapiro–Wilk, Breusch–Pagan: four of five headline taxa, seven of 12 headline pathways), and the primary and compositional (ANCOM-BC2) analyses are expected to diverge to some degree, consistent with the broader literature on method-dependent variability in microbiome differential-abundance testing [28]. Our primary method was chosen because it operates directly on the relative-abundance data MetaPhlAn4/HUMAnN3 produce, not on the assumption that it outperforms compositional-aware methods; no single differential-abundance approach performs best across all microbiome datasets. Our primary findings should be read as one well-supported account of these data, reinforced by the ANCOM-BC2 comparison above, not as a definitive result. Mock microbial communities were not included, so extraction bias could not be directly assessed. However, stool is a high-biomass specimen in which reagent contaminants contribute minimally, and the stable sequencing depth across samples argues against major technical artefacts. A substantial proportion of samples failed preparation or sequencing QC. Failure rates did not differ significantly between groups, but non-random sample loss may have introduced selection bias.

Overall, this study adds a Central Asian cohort to the literature on gut microbiome alterations in AD. Among our findings, depletion of *Faecalibacterium*, enrichment of *Eisenbergiella*, and enrichment of *Desulfovibrio* were the most consistent, remaining significant with stable effect size across the nested group/age/BMI models (Supplementary Table S1); *Eisenbergiella* and *Desulfovibrio* were additionally confirmed by the compositional ANCOM-BC2 sensitivity analysis, while *Faecalibacterium* was not, though direction was concordant throughout. More broadly, it reinforces a recurring theme: depletion of butyrate-producing *Firmicutes* and enrichment of taxa capable of sulfate reduction and hydrogen sulfide production are among the more reproducible signals across cohorts, while several other taxonomic and functional associations—including the direction of the cysteine-pathway and archaeal-pathway findings reported here relative to other functional-metagenomic studies—look cohort- or population-specific. Which of these signals reflect a shared biology of AD, and which simply reflect the microbiome’s sensitivity to geography, diet, and host genetics, is a question for larger, longitudinal, geographically diverse studies. Detailed dietary and medication data, microbial metabolomics, and amyloid/tau biomarkers would help link microbial signals more directly to AD pathology.

## 4. Materials and Methods

### 4.1. Study Design and Participants

We performed a cross-sectional case–control study comparing the gut microbiome of patients with Alzheimer’s disease (AD) and cognitively healthy controls. AD patients were recruited among residents of LLP “Nursing Care Center Meyirim”, Almaty, Kazakhstan; controls were recruited from primary care clinics, Almaty, Kazakhstan. Recruitment took place between December 2023 and December 2024. The study was approved by the Local Bioethics Committee of LLP “Kazakh Academy of Nutrition” (Protocol No. 5-2023, 15 November 2023) and conducted according to the Declaration of Helsinki. All participants, or their legal representatives where applicable, provided written informed consent before enrollment. All 35 AD patients in the final analysis were long-term residents of the nursing-care facility at the time of sample collection; AD patients who were not residents were excluded from the recruitment. Residents received standardized nutrition and followed a daily routine per the facility’s requirements, and were under routine long-term medical treatment as part of institutional care. Controls were community-dwelling and not subject to this institutional diet or treatment regimen.

**Figure 5:**
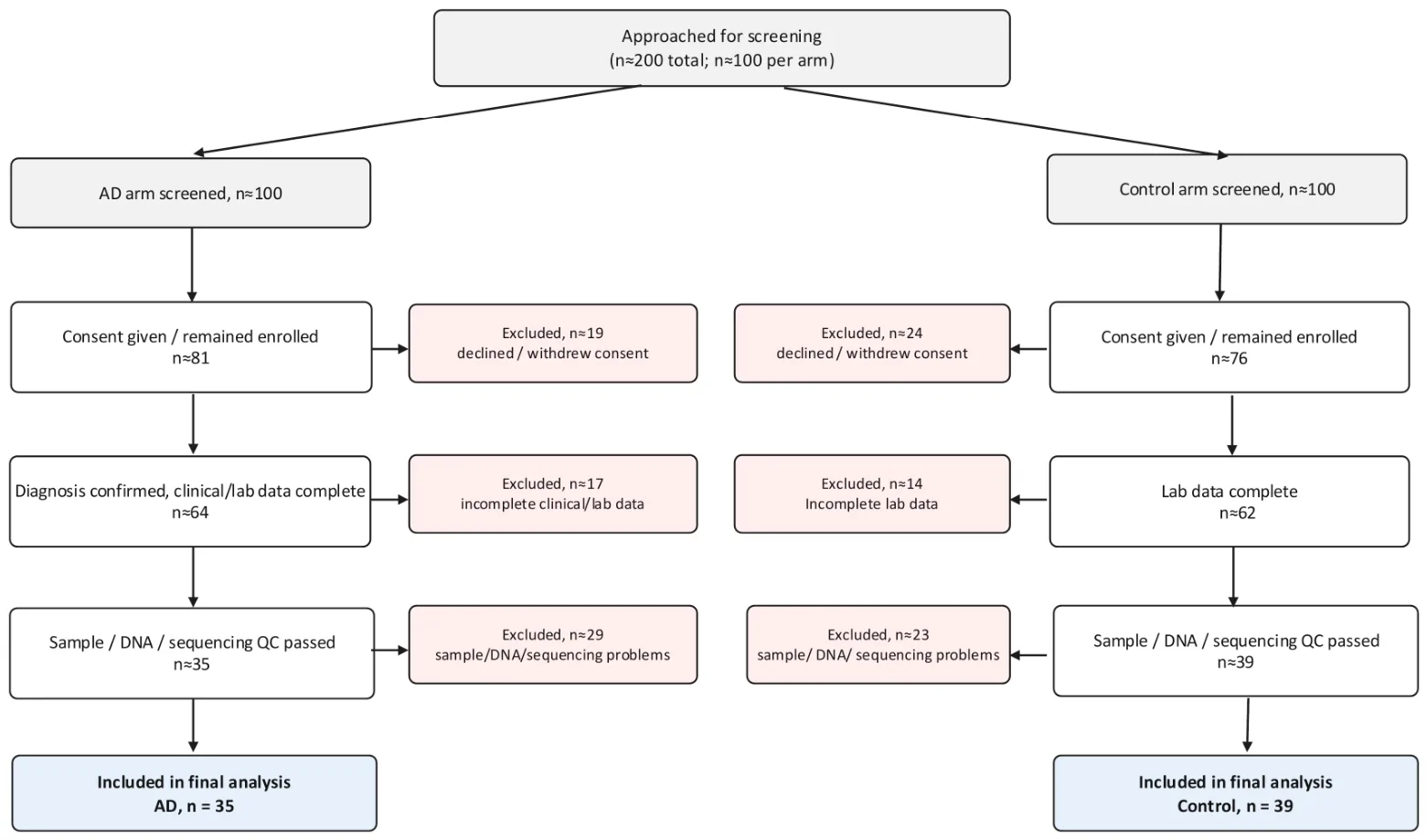
Study design and participant flow.

Of 100 individuals with a diagnosis of AD approached for screening, 35 were included in the final analysis; of 100 potential controls approached, 39 were included (Figure 5). 

Eligibility criteria were applied before individuals were approached, so no eligibility exclusions occurred within the funnel. Of those approached, 19 AD and 24 Control participants declined or withdrew consent; a further 17 AD and 14 Control patients had incomplete clinical/laboratory data; and 29 AD and 23 Control samples failed sample preparation or sequencing quality control (QC)—insufficient DNA quantity/quality for library preparation, or sequencing depth below the 6 Gb per sample. The proportion of samples failing QC did not differ significantly between groups (AD 29/64, 45.3% vs. Control 23/62, 37.1%; Fisher’s exact *p* = 0.37).

### 4.2. Inclusion and Exclusion Criteria

AD group—inclusion: clinical diagnosis of probable Alzheimer’s disease dementia per NIA-AA 2011 core clinical criteria; and age 59 years and older. All patients had a newly established diagnosis of Alzheimer’s disease at the time of enrollment. Diagnosis was based on cognitive assessment with the Montreal Cognitive Assessment (MoCA), dementia staging using WHO criteria and the Clinical Dementia Rating (CDR) scale, and structural neuroimaging (CT or MRI) where available. For patients without imaging, the Hachinski Ischemic Score—based on stroke history, cardiovascular risk factors, and pattern of decline (abrupt/stepwise vs. gradual)—was used to distinguish a vascular from a neurodegenerative contribution; cases with substantial vascular contribution were classified as mixed dementia. CSF amyloid/tau and amyloid-PET biomarkers were not available in Kazakhstan at the time of sample collection. Control group—inclusion: age 55 years and older, no subjective cognitive or memory complaints, no history of neurological or psychiatric disease, no severe somatic disease; assessed by an experienced neurologist via clinical evaluation and MMSE, with a score ≥24 required for inclusion; and able to provide informed consent directly. All cognitive assessments were performed by experienced neurologists using standardized administration of each instrument. However, the instruments differed between groups: AD patients were assessed with MoCA and CDR, whereas Controls were screened with MMSE. Cognitive scores are therefore not directly comparable between groups and were not used as covariates.

Exclusion criteria (both groups): antibiotic, probiotic, or prebiotic use within the preceding 3 months before sample collection; major gastrointestinal disease or surgery; other neurodegenerative disease or major psychiatric disorder confounding cognitive assessment; and severe systemic illness or immunosuppressive therapy.

### 4.3. Clinical and Laboratory Measurements

Venous blood samples were collected after an overnight fast from all participants at enrollment for the laboratory parameters (glucose, ALT, AST, total bilirubin, CRP, total cholesterol, HDL, LDL, triglycerides, atherogenic index, and insulin), measured using standard clinical laboratory methods; the atherogenic index was calculated as (total cholesterol—HDL)/HDL. Circulating adiponectin, measured only in the AD group, was quantified by enzyme-linked immunosorbent assay (ELISA) using a commercial human ADP/adiponectin ELISA kit (FineTest, Wuhan Fine Biotech, Wuhan, China; catalog no. EH2593, lot FN260706E), following the manufacturer’s protocol. Samples were diluted 1:100,000 before assay. The lyophilized standard was reconstituted and serially diluted to generate a seven-point calibration curve (15.625–1000 pg/mL, with sample diluent as the zero standard) run on each plate (R^2^ ≥ 0.99); absorbance was read at 450 nm. Because of limited biospecimen volume, samples were assayed in singlicate, with repeat testing at an adjusted dilution for values outside the calibration range. Manufacturer-reported intra- and inter-assay coefficients of variation were ≤6%.

### 4.4. Sample Collection, DNA Extraction, and Sequencing

Fecal specimens were collected using Zymo Research DNA/RNA Shield™ (Zymo Research, Irvine, CA, USA) collection tubes, with participants given detailed written and verbal instructions for proper sample collection and storage; specimens were transported to National Laboratory Astana within 24 h of collection and immediately cryopreserved at −80 °C pending analysis. Microbial DNA was extracted from fecal samples using the ZymoBIOMICS DNA Miniprep Kit (Cat# D4300, Zymo Research, Irvine, CA, USA) according to the manufacturer’s protocol; DNA quality and concentration were assessed via spectrophotometry NanoDrop 2000 (Thermo Fisher Scientific, Waltham, MA, USA), fluorometry Qubit 3.0 (Thermo Fisher Scientific, Waltham, MA, USA), and agarose gel electrophoresis. DNA extraction was performed in order of sample receipt. Samples with a DNA yield of ≥50 ng proceeded to library preparation. Shotgun metagenomic libraries (short-insert, paired-end 150 bp) were prepared within a single run and sequenced by BGI Genomics on the DNBSEQ platform (BGI Genomics, Shenzhen, China) using the same protocol for all samples. Samples yielding <6 Gb of clean data after filtering were excluded. Retained samples had a mean sequencing depth of 23.9 ± 0.6 million read pairs per sample (range 21.1–24.2 million; mean Q30 94.4%, mean GC content 47.8%), indicating uniform depth across samples.

### 4.5. Bioinformatic Analysis

Raw sequencing reads underwent adapter and quality filtering using SOAPnuke (v2.1.9), (parameters: -*n* 0.01 -l 20 -q 0.5 --adaMis 3 --minReadLen 150). Human host reads were removed by alignment to the human reference genome (GRCh38) using Bowtie2 (v2.4.4); the proportion of human reads was low (mean 0.27%, range 0.08–0.67%). Taxonomic profiling was performed with MetaPhlAn 4.0.3 using the database mpa_vJan21_CHOCOPhlAnSGB_202103. Functional and pathway profiling was performed with HUMAnN 3.6.1 (ChocoPhlAn v201901_v31 and UniRef90 v201901b, EC-filtered databases), using the MetaPhlAn 4 taxonomic profile as input.

### 4.6. Statistical Analysis

Statistical analyses were performed in Python (v3.9.13) using pandas (v2.3.3), NumPy (v1.24.4), SciPy (v1.9.1), and statsmodels (v0.14.6); supplementary tables were built with openpyxl (v3.1.5), and figures were generated with Matplotlib (v3.5.2). Permutation-based tests (PERMANOVA, PERMDISP, marginal PERMANOVA) used a fixed random seed (seed = 0, 999 permutations) for exact reproducibility.

Unless otherwise noted, two-group comparisons of continuous variables throughout this study—demographic/clinical parameters, alpha diversity, and differential abundance of taxa and metabolic pathways—were performed using the two-sided Mann–Whitney U test; categorical variables were compared using Fisher’s exact test or the chi-square test.

Alpha diversity was assessed at the species level using observed richness, the Shannon index, the Simpson index, and Pielou’s evenness, together with the Firmicutes/Bacteroidetes ratio computed at the phylum level. These five metrics were compared between groups (Mann–Whitney U, as above) with Benjamini–Hochberg false discovery rate (FDR) correction, unadjusted and, after z-scoring each metric, adjusted for age and BMI using ordinary least squares (OLS) regression of the z-scored metric on group, age and BMI, with the group-coefficient *p*-value FDR-corrected across the five metrics. Beta diversity was assessed on genus-level relative abundances (prevalence ≥ 10%), Hellinger-transformed and compared using Bray–Curtis dissimilarity; group separation was visualized by principal coordinate analysis (PCoA). The unadjusted group effect was tested by PERMANOVA (scikit-bio, 999 permutations), with PERMDISP used to confirm homogeneity of group dispersions [29,30]. The age/BMI-adjusted group effect, and the corresponding age effect adjusted for group and BMI, were tested by partial PERMANOVA (sequential sum-of-squares, 999 permutations), with the term of interest entered last so its sum of squares reflects its contribution beyond the other covariates. Individual clinical and demographic variables were separately screened for association with community composition using marginal (univariate) PERMANOVA, with BH-FDR correction applied across variables. Family-level relative abundances (≥70% prevalence in either group) were summarized per sample, with less prevalent families pooled as “Other”. Adiponectin, measured only in the AD group, was tested separately within AD patients and excluded from the FDR correction above.

Differential abundance of individual taxa and of HUMAnN-derived metabolic pathways (filtered to ≥10% prevalence) between AD and Control groups was assessed as above, with BH-FDR correction (*q* < 0.05), unadjusted and after adjustment for age and BMI (OLS regression on arcsine-square-root-transformed abundance). This approach was chosen because it operates directly on the relative-abundance output of MetaPhlAn4/HUMAnN3 without requiring pseudo-absolute-abundance reconstruction; performance differences between it and compositional-aware methods on real microbiome data at this sample size are not well established [10], so we validated it directly against ANCOM-BC2 (see below) rather than relying on method choice alone. As a compositional sensitivity analysis, pseudo-absolute-abundance tables were reconstructed from each sample’s MetaPhlAn4/HUMAnN3 relative abundance (expressed as a fraction of 1) multiplied by that sample’s total post-QC read count, rounded to the nearest integer. This reconstruction was used because ANCOM-BC2 expects count data, not relative abundances. It assumes sequencing depth roughly reflects total microbial load, which is an approximation, not the true absolute abundance. ANCOM-BC2 (scikit-bio v0.7.4 ancombc2, matching the R ANCOMBC algorithm) was applied with unadjusted (~group) and age + BMI-adjusted (~age + bmi + group) models, a pseudocount of 1, and BH-FDR correction within each taxonomic rank and within pathways. Age and BMI differed significantly between groups and were used as covariates for this reason. We additionally fit nested models (group-only, +age, +age + BMI) for the taxa and pathways discussed in greatest detail below.

Effect size for pathways was log2 fold-change (AD/Control) with 95% bootstrap confidence intervals: 5000 resamples with replacement, drawn independently within each group, recomputing the group mean each time. Zero values were handled with a pseudocount (half the smallest non-zero value in the matrix), added before the log2 transform. Pathways were classified by MetaCyc functional category. Differentially abundant taxa were visualized as cladograms using the LEfSe plotting utility (lefse_plot_cladogram.py [31]), repurposed here for tree rendering only: node color and the accompanying legend reflect the group-wise direction and *q*-values from our own Mann–Whitney U/BH-FDR analysis described above.

Associations between the top differentially abundant taxa and pathways and 14 individual clinical and demographic variables (age, BMI, sex, and 11 laboratory parameters—glucose, insulin, ALT, AST, total bilirubin, CRP, total cholesterol, HDL, LDL, triglycerides, and atherogenic index; all z-scored) were tested using OLS regression on arcsine-square-root-transformed relative abundance, adjusting for diagnosis group, with BH-FDR correction applied within each clinical variable × domain (taxa, pathways) block. Diagnosis group itself and ethnicity (a multi-category variable not reducible to a single regression coefficient) were not included in this panel; both were instead assessed in the 16-variable community-composition screen described above. Standardized coefficients were computed as the raw OLS coefficient (per 1 SD of the z-scored clinical variable) divided by the standard deviation of the arcsine-square-root-transformed response.

Because adiponectin was measured only in the AD group, its association with gut microbiome composition and function was tested separately within AD patients using Spearman’s rho, with BH-FDR correction applied within each domain. *q* < 0.05 (*) and *q* < 0.01 (**) were considered significant. This screen comprised 742 tests across 28 independently-controlled FDR families (14 variables × 2 domains), plus a 277-test, 3-domain screen for adiponectin; we additionally applied a single BH-FDR correction across all 1019 tests as a global sensitivity check. Given the sample size and number of comparisons performed, group-wise differential abundance testing (taxa, pathways, alpha/beta diversity) constitutes the primary, BH-FDR-corrected analysis; the clinical/demographic correlation screen and the adiponectin analysis are exploratory, hypothesis-generating, and not intended to support independent causal claims.

Taxon-stratified contributions to individual pathways were extracted from per-sample HUMAnN output where available. Spearman correlation, BH-FDR corrected across these comparisons, additionally tested, within the AD group, pairwise relationships among the top differentially abundant taxa.

## 5. Conclusions

Overall, this study adds a Central Asian cohort to the literature on gut microbiome alterations in AD. Among our findings, depletion of *Faecalibacterium,* enrichment of *Eisenbergiella*, and enrichment of *Desulfovibrio* were the most consistent, remaining significant with stable effect size across the nested group/age/BMI models (Supplementary Table S1); *Eisenbergiella* and *Desulfovibrio* were additionally confirmed by the compositional ANCOM-BC2 sensitivity analysis, while *Faecalibacterium* was not, though direction was concordant throughout. More broadly, it reinforces a recurring theme: depletion of butyrate-producing Firmicutes and enrichment of taxa capable of sulfate reduction and hydrogen sulfide production are among the more reproducible signals across cohorts, while several other taxonomic and functional associations look cohort- or population-specific, including the direction of the cysteine-pathway and archaeal-pathway findings reported here relative to other functional-metagenomic studies. Which of these signals reflect a shared biology of AD, and which simply reflect the microbiome’s sensitivity to geography, diet, and host genetics, is a question for larger, longitudinal, geographically diverse studies. Detailed dietary and medication data, microbial metabolomics, and amyloid/tau biomarkers would help link microbial signals more directly to AD pathology.

## Figures and Tables

**Table 1:** Demographic and clinical parameters of the study groups.

Parameter	AD (*n* = 35)	Control (*n* = 39)	*p*-Value	Test
**Demographic parameters**				
Sex, male	9 (25.71%)	9 (23.08%)	*p* = 1.0000	Fisher’s exact
Sex, female	26 (74.29%)	30 (76.92%)	*p* = 1.0000	Fisher’s exact
Age, years	82.00 (IQR: 74.00; 87.50)	67.00 (IQR: 61.00; 71.50)	*p* < 0.0001 *	Mann–Whitney U rank test
BMI, kg/m^2^ (*n* = 35/39)	23.10 (IQR: 21.70; 25.20)	26.49 (IQR: 23.63; 28.98)	*p* = 0.0014 *	Mann–Whitney U rank test
Ethnicity, Kazakh	11 (31.43%)	22 (56.41%)	*p* = 0.0878	Chi-square
Ethnicity, Other	3 (8.57%)	3 (7.69%)		
Ethnicity, Russian	21 (60.00%)	14 (35.90%)		
**Laboratory parameters**				
Glucose, mmol/L (*n* = 35/39)	4.40 (IQR: 4.15; 4.98)	5.39 (IQR: 4.85; 5.87)	*p* = 0.0001 *	Mann–Whitney U rank test
ALT, U/L (*n* = 35/39)	8.80 (IQR: 6.50; 14.05)	13.05 (IQR: 7.70; 18.40)	*p* = 0.0975	Mann–Whitney U rank test
AST, U/L (*n* = 35/39)	16.80 (IQR: 13.40; 18.90)	18.10 (IQR: 15.45; 19.95)	*p* = 0.0832	Mann–Whitney U rank test
Total bilirubin, µmol/L (*n* = 35/39)	9.40 (IQR: 6.95; 12.75)	8.30 (IQR: 6.30; 10.90)	*p* = 0.1674	Mann–Whitney U rank test
CRP, mg/L (*n* = 35/39)	1.47 (IQR: 0.65; 2.21)	0.95 (IQR: 0.60; 3.25)	*p* = 0.6222	Mann–Whitney U rank test
Total cholesterol, mmol/L (*n* = 35/39)	5.13 (IQR: 4.14; 5.59)	5.42 (IQR: 4.51; 5.92)	*p* = 0.0681	Mann–Whitney U rank test
HDL, mmol/L (*n* = 35/39)	1.30 (IQR: 1.17; 1.48)	1.42 (IQR: 1.04; 1.65)	*p* = 0.4917	Mann–Whitney U rank test
LDL, mmol/L (*n* = 35/39)	3.24 (IQR: 2.41; 3.50)	3.19 (IQR: 2.91; 3.63)	*p* = 0.1561	Mann–Whitney U rank test
Triglycerides, mmol/L (*n* = 35/39)	1.29 (IQR: 1.02; 1.67)	1.08 (IQR: 0.90; 1.73)	*p* = 0.4261	Mann–Whitney U rank test
Atherogenic index (*n* = 35/39)	2.70 (IQR: 2.05; 3.40)	2.95 (IQR: 2.15; 3.35)	*p* = 0.6374	Mann–Whitney U rank test
Insulin, µIU/mL (*n* = 35/39)	4.57 (IQR: 2.81; 8.03)	8.90 (IQR: 6.25; 11.70)	*p* = 0.0009 *	Mann–Whitney U rank test
Adiponectin, µg/mL (*n* = 35/0)	48.31 (IQR: 34.23; 71.66)	–	–	–

* *p* ≤ 0.05 (raw, unadjusted). Atherogenic index = (total cholesterol—HDL)/HDL. Unlike microbiome taxa/pathway *q*-values in this study, no multiple-testing correction was applied here, per standard practice for baseline characteristics.

## Data Availability

The raw sequencing data are available at the National Center for Biotechnology Information (NCBI) Sequence Read Archive (SRA) under BioProject accession number PRJNA1520107. Processed data supporting the findings are provided in Supplementary Tables S1–S5. Analysis code is available from the corresponding author upon reasonable request.

## Supplementary Materials

The following supporting information can be downloaded at https://www.mdpi.com/article/10.3390/ijms27219543/s1.
